# Mitochondrial genetic differentiation across populations of the malaria vector *Anopheles lesteri *from China (Diptera: Culicidae)

**DOI:** 10.1186/1475-2875-10-216

**Published:** 2011-08-03

**Authors:** Manni Yang, Yajun Ma, Jing Wu

**Affiliations:** 1 Department of Pathogen Biology, Second Military Medical University, Shanghai 200433, China

## Abstract

**Background:**

*Anopheles lesteri *is a primary vector of *Plasmodium *spp. in central China. A complete understanding of vector population structure and the processes responsible for the differentiation is important to the vector-based malaria control programmes and for identifying heterogeneity in disease transmission as a result of discrete vector populations. There is no adequate *An. lesteri *population genetic data available.

**Methods:**

Polymorphism of sequence variations in mitochondrial COII and Cytb genes were assessed to explore the level of genetic variability and differentiation among six populations of *An. lesteri *from China.

**Results:**

There were 30 (4.37%) and 21 (5.33%) polymorphic sites for mtDNA-COII and Cytb gene, respectively. Totally 31 COII and 30 Cytb haplotypes were obtained. The range of *F_ST _*values was from 0.101 to 0.655 by mtDNA-COII, and 0.029 to 0.231 by Cytb gene. The analysis of molecular variance (AMOVA) showed that the percentage of variation within populations (65.83%, 88.48%) was greater than that among populations (34.17%, 11.52%) using both genes. The Tajima's *D *and Fu's *Fs *values were all negative, except Tajima's *D *values of YN and HNB populations, which suggest a large number of low-frequency mutations in populations and the populations were in expansion proceeding.

**Conclusions:**

Levels of genetic variation within *An. lesteri *populations were higher than among them. While these results may suggest considerable levels of gene flow, other explanations, such as the effect of historical population perturbations can also be hypothesized.

## Background

*Anopheles lesteri*, which belongs to the Hyrcanus group of the genus *Anopheles *is a primary vector of malaria in central China [1]. Genetically-based methods have been proposed for malaria vector control. These methods focus mainly in altering vectorial capacity through the genetic modification of natural vector populations by means of introducing refractoriness genes or by sterile insect technologies [2]. Knowledge of the genetic structure of vector species is, therefore, an essential requirement as it should contribute not only to predict the spread of genes of interest, such as insecticide resistance or refractory genes, but also to identify heterogeneities in disease transmission due to distinct vector populations [3]. A complete understanding of vector population structure and the processes responsible for the distribution of differentiation is important to vector-based malaria control programmes and for identifying heterogeneity in disease transmission as a result of discrete vector populations [4]. Susceptibility to *Plasmodium *infection, survival and reproductive rates, degree of anthropophily, and the epidemiology of malaria in the human host may all be affected by genetic variation in vector populations [5].

*Anopheles lesteri *is almost morphologically undistinguishable from its sibling species because of lacking the objective and stable identification characters, so the taxonomic status on *An. lesteri *in China has revised many times. Xu and Feng [6] regarded the Chinese "*An. lesteri*" as a new subspecies *An. lesteri anthropophagus *because it was distinct from both *An. lesteri lesteri *from the Philippines and *An. lesteri paraliae *[7] from Malaysia in bionomics as well as morphology. The subspecies was later elevated to a full species rank [8]. However, the second internal transcribed spacer (ITS2) of ribosomal DNA (rDNA) of *An. anthropophagus *in China was similar to that of *An. lesteri *from the Philippines, South Korea, Guam and Japan [9,10]. The molecular evidence strongly support that *An. anthropophagus *is the synonym of *An. lesteri*.

*Anopheles lesteri *exhibits variation in ecology [11], morphology [12], chromosomes [12], and random amplified polymorphic DNA (RAPD) markers [13]. Furthermore, *An. lesteri *was not considered as malaria vector in Guam and Philippines, but had high transmission capacity of malaria in central China [11,14], and a certain transmission capacity in South Korea and Japan [15,16]. Despite its significance in malaria transmission, only a few studies on population genetics have been conducted [13]. Many genes of mtDNA were used to analyse the genetic variation and population structure of the Anopheline mosquitoes, such as cytochrome subunit I (COI) [17-20], cytochrome subunit II (COII) [21,22], control region [23], NADH dehydrogenase subunit 4 [24] and subunit 5 [4,25-29]. The present study aimed to estimate genetic variability and population structure and to infer the extent of gene flow among *An. lesteri *populations from China based on mtDNA-COII and cytochrome B (Cytb) genes sequences.

## Methods

### Mosquito collections and species identification

**Figure 1:**
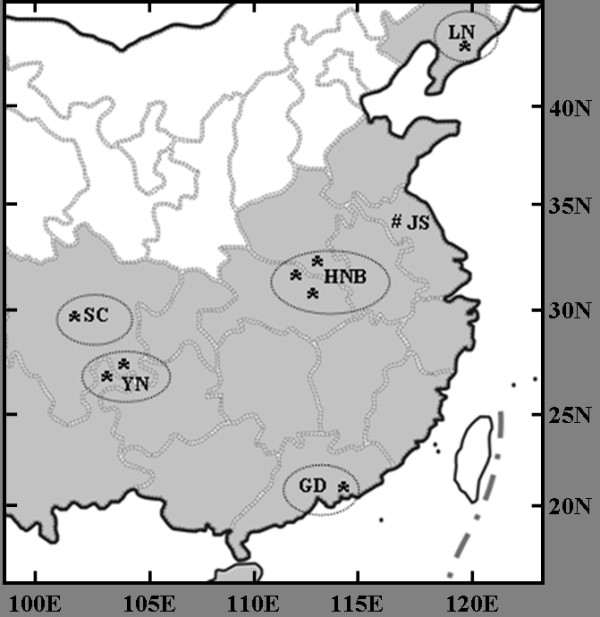
**Localities of collections for populations of *Anopheles lesteri *in China**. The places in grey indicate the distribution areas. The five field populations are enclosed in separate circles. The well code was the original collection site of the JS lab colony.

Wild adult *An. lesteri *were collected from 2004 to 2007, by using indoor light traps and human landing catches at human living room and livestock corrals. The eight collection sites in China were located from 22°17'N to 39°58'N, and 103°29'E to 123°50'E (Table 1 Figure 1). The HNB and YN populations consisted of specimens pools from two or three sites in proximity to each other, as stated in Table 1. The distances between sites were below 50 km. There were total five field populations and a laboratory colony, with JS population in this study.

**Table 1:** The collecting data of *Anopheles lesteri *mosquito populations in this study

Population Code	Collecting site	Collecting date	Sample size	Latitude (N)	Longitude (E)
GD	Zhuhai, Guangdong	Oct. 2007	22	22°17'	113°30'
YN	Yanjing, Yunnan	June 2006	9	28°60'	104°13'
	Junlian, Sichuan	June 2006	4	28°10'	104°34'
SC	Pujiang, Sichuan	June 2006	23	30°14'	103°29'
HNB	Guangshui & Suizhou, Hubei	June 2007	8	31°41'-31°52'	113°15'-113°47'
	Tongbai, Heinan	June 2007	5	32°29'	113°23'
JS*	Xuyi, Jiangsu	June 1985	17	32°54'	118°34'
LN	Donggang, Liaoning	June 2004	28	39°58'	123°50'

* The collecting site and date of laboratory colony were original information. The mosquitoes were kept at 26 ± 1°C and 65 ± 5% (RH), under a 12: 12 hr (light: dark) photoperiod.

Adult mosquitoes of *An. hyrcanus *group were identified by morphology using the identification keys of Lu *et al *[14]. Specimens were kept individually in silica gel filled tubes at 4°C, until DNA extraction was performed according to Collins *et al *[30]. *Anopheles lesteri *species identification was done by a PCR assay based on rDNA-ITS2 markers previously described in Ma *et al *[31].

### mtDNA-COII and Cytb genes amplification and sequencing

Sequence variation was examined in the mtDNA-COII and the Cytb genes. The COII and Cytb regions were amplified in 50 μL reaction mixtures containing 1 × reaction buffer (QIAGEN, Courtaboeuf, France), 0.1 mM of each dNTP (Eurogentec, Angers, France), 1 unit of *Taq *DNA polymerase, 0.1 μM each of the forward and reverse primers and 1.5 μL genomic DNA. The COII gene was amplified using primers COIIF (5'- TCT AAT ATG GCA GAT TAG TGC A -3', forward) and COIIR (5'- ACT TGC TTT CAG TCA TCT AAT G -3', reverse), and the Cytb gene using primers CytbF (5'- GGA CAA ATA TCA TTT TGA GGA GCA ACA G-3', forward) and CytbR (5'- ATT ACT CCT CCT AGC TTA TTA GGA ATT G -3', reverse). The cycle conditions in PTC-100 Peltier Thermal Cycler included an initial denaturation step at 94°C for 2 min, followed by 30 cycles at 94°C for 30 s, 50°C for 30 s and 72°C for 30 s, with a final extension step at 72°C for 8 min. After electrophoresis, PCR products were purified and used for sequencing in both directions with the previous primers, on an ABI 3730 automatic sequencer (Applied Biosystems). Sequences were inspected and corrected, where necessary, using SEQSCAPE software (Applied Biosystems).

### Data analyses

Multiple sequence alignments for each gene were performed using MEGA 4.0 [32] and CLUSTAL × [33]. The sequences polymorphism was assessed with MEGA 4.0. A haplotype networks and outgroup probability of the haplotypes were constructed based on statistical parsimony using TCS 1.21 [34].

The parameters θ_π _equivalent to the average pairwise number of differences between sequences [35], θ_s _equivalent to the number of segregating nucleotide sites per sequence [36], and haplotypes diversity (*h*) were estimated for COII and Cytb polymorphism within populations. The population genetic structure was analysed with 5 field populations, and assessed by analyzing molecular variance with ARLEQUIN 3.11 [37]. The percentage of sequence divergence within and between populations was calculated based on Nei and Li [38], and pairwise *F_ST _*values for short-term genetic distance between populations were estimated with the methods of Slatkin (1995) [39] and tested for significance by permutation. Mismatch distributions were calculated using ARLEQUIN 3.11, and the neutrality tests were evaluated by Tajima's *D *and Fu's *Fs*. Isolation by geographical distance was assessed by GENEPOP 4.0.10 [40] using Mantel test.

## Results

### Sequences characteristics of mtDNA-COII

**Figure 2:**
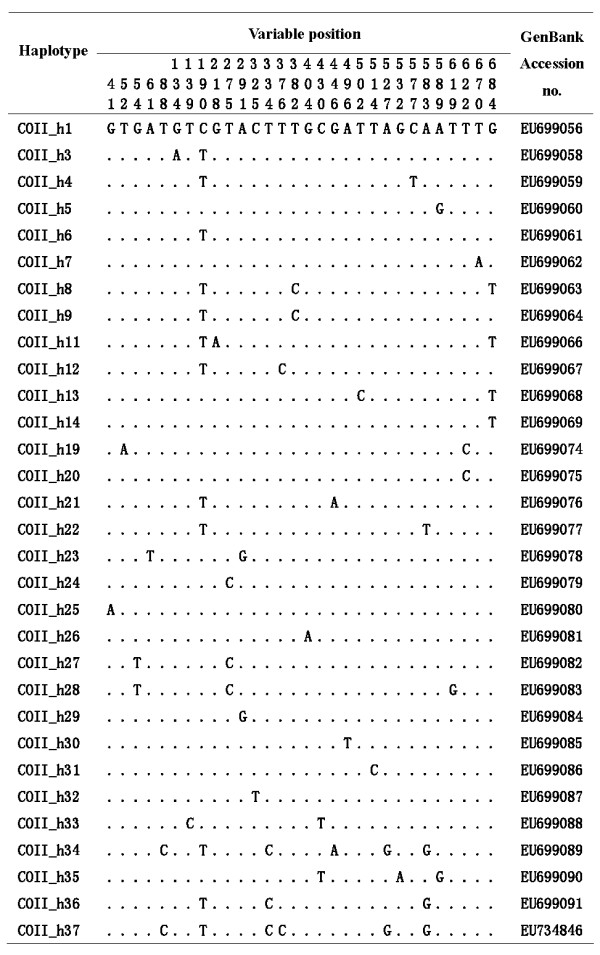
**Variable bases of the mtDNA-COII gene for the haplotypes of *Anopheles lesteri *population**.

**Figure 3:**
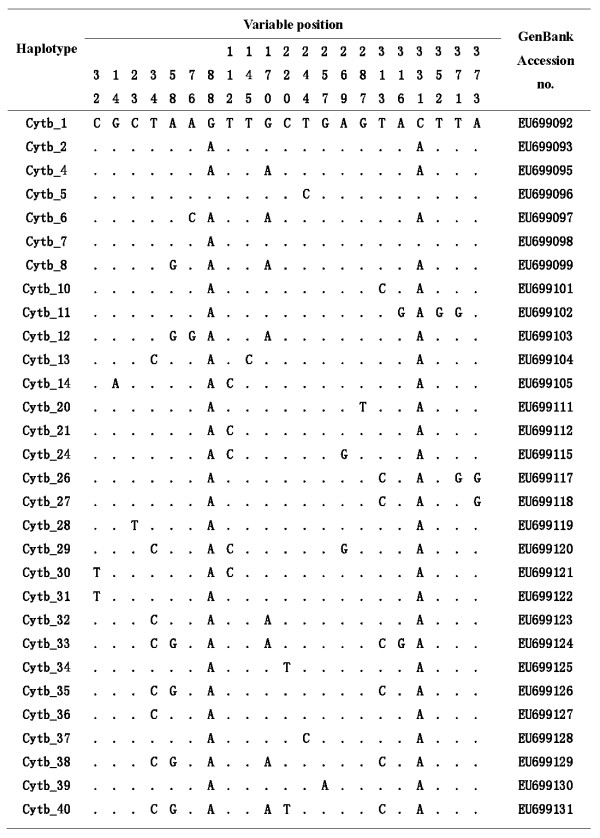
**Variable bases of the mtDNA-Cytb gene for the haplotypes of *Anopheles lesteri *population**.

One hundred and sixteen *An. lesteri *mosquitoes were distinguished by PCR assay from China (Table 1). A 686 bp COII sequence was determined in 88 mosquitoes, and a Cytb fragment of 394 bp was obtained from 112 mosquitoes. All segregating sites and the sequence variants (haplotypes) are shown in Figures 2 and 3. The summary statistics for both genes are given in Table 2. Across the whole dataset, there were 30 (4.37%) and 21 (5.33%) polymorphic sites for COII and Cytb, respectively. This low number of variable sites resulted in low nucleotide diversity and low haplotype diversity across samples. The θ_S _of overall field populations was from 0.581 ± 0.435SD to 4.285 ± 1.709SD for COII, and 0.274 ± 0.274SD to 3.545 ± 1.655SD for Cytb; θ_π _was from 0.477 ± 0.485SD to 2.598 ± 1.606SD for COII, 0.091 ± 0.188SD to 2.231 ± 1.476SD for Cytb and *h *was from 0.005 ± 0.003SD to 0.000 ± 0.000SD (Table 2).

**Table 2:** Data summary for populations, haplotypes and nucleotide diversity of *Anopheles lesteri*

Population	Gene	N	Haplotypes (n)	*S*	*h *(± SD)	θ_s _(± SD)	θ_π _(± SD)
YN	COII	8	**1(2)**, 3(2), 4(2), **5(1)**, **6(1)**	4	0.002 ± 0.002	1.543 ± 0.961	1.643 ± 1.227
	Cytb	13	**1(2)**, **2(5)**, **4(3)**, 5(1), 6(1), 7(1)	5	0.004 ± 0.003	1.611 ± 0.899	1.615 ± 1.147
HNB	COII	8	**1(1)**, 8(1), 9(1), 11(1), 12(2), 13(1), 14(1)	8	0.004 ± 0.002	2.314 ± 1.308	2.464 ± 1.692
	Cytb	12	**1(3)**, **2(2)**, **4(1)**, 8(1), 10(1), 11(1), 12(1), 13(1), 14(1)	13	0.006 ± 0.004	3.545 ± 1.655	2.231 ± 1.476
JS	COII	12	19(2), **20(10)**	1	0.000 ± 0.002	0.331 ± 0.331	0.303 ± 0.379
	Cytb	17	**2(17)**	0	0.000 ± 0.000	0.000 ± 0.000	0.000 ± 0.000
SC	COII	18	**6(3)**, 21(14), 22(1)	2	0.001 ± 0.001	0.581 ± 0.435	0.477 ± 0.485
	Cytb	22	**2(21)**, **4(1)**	1	0.000 ± 0.000	0.274 ± 0.274	0.091 ± 0.188
GD	COII	18	**1(9)**, 23(1), 24(3), 25(1), 26(1), 27(1), 28(1), 29(1)	7	0.002 ± 0.001	2.035 ± 1.006	1.288 ± 0.946
	Cytb	21	**2(6)**, **4(2)**, 20(1), 21(2), 24(2), 26(1), 27(3), 28(1), 29(1), 30(1), 31(1)	10	0.005 ± 0.003	2.780 ± 1.239	2.076 ± 1.350
LN	COII	24	**1(4)**, **5(1)**, 7(1), **20(8)**, 30(1), 31(1), 32(3), 33(1), 34(1), 35(1), 36(1), 37(1)	15	0.005 ± 0.002	4.285 ± 1.709	2.598 ± 1.606
	Cytb	27	**2(14)**, **4(1)**, 32(1), 33(1), 34(1), 35(3), 36(1), 37(1), 38(2), 39(1), 40(1)	9	0.005 ± 0.003	2.037 ± 0.928	1.926 ± 1.255

*h *is haplotype diversity, *S *is the number of segregating sites and θ_s _and θ_π _are the estimates of nucleotide diversity. The bold haplotypes occur in more than one population and the number in parentheses indicates the frequency of the haplotype.

**Figure 4:**
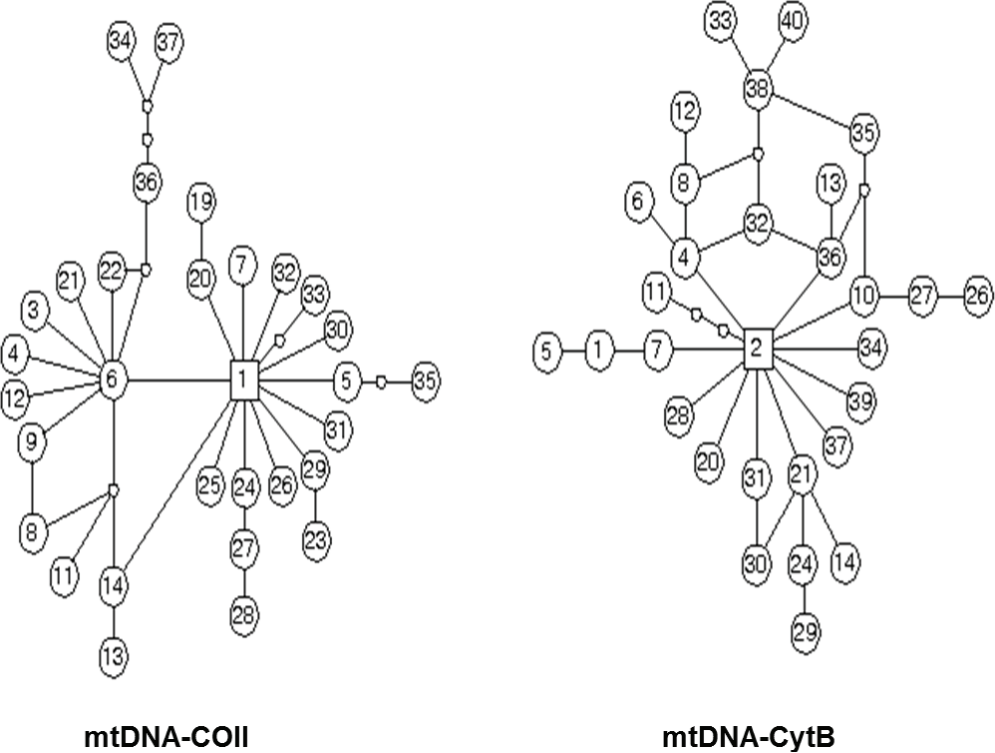
**Genealogical relationships among haplotypes of two mtDNA genes estimated by TCS**. The circle indicates one of haplotypes and the number in circle represents the type of haplotype. A unit branch represents one mutation. The empty circles indicate haplotypes that were not observed.

Among the 88 COII sequences, 31 haplotypes were found. Four haplotypes of COII_1, COII_5, COII_6 and COII_20 occurred in more than one population, the frequency was 12.90% (4/31). Thirty of 112 Cytb haplotypes were observed. Three haplotypes of Cytb_1, Cytb_2 and Cytb_4 were shared, especially; Cytb_2 occurred in all populations (Table 2). Haplotype networks showed that *An. lesteri *haplotypes derived from a single common ancestral COII haplotype and two ancestral Cytb haplotypes (Figure 4).

### Population genetic structure of *An. lesteri *population

The genetic structure was analysed with GD, LN, YN, SC and HNB populations. The range values of paiwise *F_ST _*was from 0.101 (GD/LN) to 0.655 (GD/SC) with mtDNA-COII, and 0.029 (HNB/LN) to 0.231 (YN/SC) with Cytb (Table 3). A Mantel test was carried out, and the correlation coefficient for the *F_ST _*with geographical distance was 0.271 by COII (*P *≥ 0.803) and 0.089 by Cytb (*P *≥ 0.400), which was not significance based on 1,000 permutations.

**Table 3:** Pairwise genetic distance (*F_ST_*) for populations of *Anopheles lesteri*

	YN	HNB	SC	GD	LN
YN	**0.345**/0.114	0.055	0.231*	0.128*	0.153*
HNB	**0.124**	**0.324**/0.096	0.125*	0.055*	0.029
SC	0.500*	0.501*	**0.374**/0.159	0.109*	0.172*
GD	0.272*	0.319*	0.655*	**0.252**/0.099	0.117*
LN	0.135*	0.194*	0.455*	0.101*	**0.315**/0.103

The pairwise values calculated by mtDNA-COII and Cytb gene are below and above the diagonal. The numbers along the diagonal are *F_ST _*values within population. The bold values are by COII gene. *P < 0.05.

In the hierarchical AMOVE, both the 'among populations' and 'within populations' variance components were considerable high, the latter was more contribution to total variances than the former (Table 4). The mean genetic divergence among populations was greater by COII (0.342) than Cytb (0.115).

**Table 4:** AMOVA analysis of genetic variation in *Anopheles lesteri *populations by mtDNA genes

Source of variation	Degree of freedom		Variance components		Percentage of variation	
	
	COII	Cytb	COII	Cytb	COII	Cytb
Among populations	4	4	0.433	0.109	34.17	11.52
Within populations	71	93	0.835	0.772	65.83	88.48
Total	75	97	1.268	0.872	100	100

*F_ST _*= 0.342(COII), 0.115 (Cytb)

The simulated mismatch distribution among the mtDNA-COII and Cytb haplotypes was smooth and unimodal peak, which coincide with the population expansion model. Although, observed value appeared multimodal, the result of variance test indicated the degree of coincidence between them was not significance (*P *≥ 0.00 with COII, *P *≥ 0.15 with Cytb) [41]. The Tajima's *D *and Fu's *Fs *values were all negative, except Tajima's *D *values of YN and HNB populations (Table 5), which suggested a large number of low-frequency mutations in populations and the populations were in expansion proceeding. The strongly negative values for Fu's *Fs *suggested population growth and this is supported by the estimated values using COII gene from the rapid expansion model fitted in ARLEQUIN (τ = 2u*t *= 2.615, θ_0 _= 0.00-0.39, θ_1 _= 99 999, u = per sequence mutation rate, *t *= time since expansion, *N *= effective number of females). With a mutation rate of 1 × 10^-8 ^per site per generation [42], these values suggested a change in population size from a few thousand females to 10^8 ^females, in the range of 3970 years ago based on two generations of Anopheline mosquitoes in one month.

**Table 5:** Values of neutrality test for *Anopheles lesteri *populations by mtDNA genes

		YN	HNB	SC	GD	LN
Tajima's *D*	COII	0.283	0.303	-0.438	-1.246*	-1.402*
	Cytb	0.009	-1.498*	-1.162	-0.873	-0.167
Fu's *Fs*	COII	-8.139	-∝	-28.504	-26.580	-26.580
	Cytb	-18.044	-15.265	-∝	-27.052	-27.112

*P < 0.1.

## Discussion

Sampling strategy and geographic coverage greatly influence the analysis and interpretation of the data generated from the samples. In China, *An. lesteri *was distributed in a range as the east of 100° E, and from 19° N to 42° N [14]. In this study, *An. lesteri *mosquitoes were collected from most localities across its range. Although field *An. lesteri *specimen was difficult to collect due to usage of insecticide and environment changes, our sampling still covered geographic span of *An. lesteri *distribution. The LN was at the most northern limit, and GD was at the most southern limit of the distribution basically.

In this study, both level of mtDNA- OII and Cytb gene nucleotide diversity in field populations were greater than JS laboratory colony, such as all Cytb sequences in JS population were the same, which was similar to other gene on mitochondrial DNA, as COI (*An. dirus, An. darlingi, An. stephensi*) [17-20] and COII (*An. jeyporiensis, An. minimus *) [21,22]. Thus, they are useful marker for exploring *An. lesteri *population genetic structure.

The pairwise genetic distance using mtDNA-COII gene (0.101-0.655) was higher than Cytb (0.029-0.231). In theory, it was hard to prevent genetic divergence caused by genetic drift if the gene flow [*Nm*= (1- *F_ST_*)/4 *F_ST_*]) value was less than one [43]. The level of gene flow in these *An. lesteri *pairwise populations was below one, except YN/HNB, YN/LN, HNB/LN and LN/GD using mtDNA-COII gene, but all more than one except SC/YN using Cytb. The shallow population genetic structure was showed by Cytb gene. But the results by COII gene suggested that there was an apparent segregation from LN with the other populations, which is in agreement with the previous investigations with RAPD markers [13]. So, the level of *An. lesteri *population genetic divergence using mtDNA-COII gene should represent wild populations.

The factors responsible for population genetic structure should be analysed related with the climate, geography and the behaviour of mosquitoes. Yunnan is a highly complex region topographically due to its transitional position from tropical southern Himalayas to eastern Asia and from tropical Southeast Asia to sub-tropical China as well as at the junction of the India and Burmese plates, derived from Gondwanaland, and the Eurasian plate [44]. It is a noted centre of biodiversity [45-47]. It could have retained sufficiently mesic habitats for mosquitoes during the glaciations, when drier, more open habitats were spread widely [48]. If YN population of *An. lesteri *was the ancestor and the other region populations spread from Yunnan in the late stage of glaciations. The haplotype network suggested that *An. lesteri *migrated and spread from Yunnan towards the North and the East China, and occurred colonization and expansion during migration proceeding. They were the same as the *An. lesteri *population patterns with *An. dirus *complex in Southeast Asia by mtDNA-COI and microsatellite DNA [17,49], *An. jeyporiensis *in South China by mtDNA-COII [21]. If the migrating and expansion route was true, the *An. lesteri *samples in south of Yunnan should be increase to further investigation. *An. lesteri *is widespread in Palaearctic and Oriental region, and there is different climate, breeding habitation and blood preference, such as *An. lesteri *in southern and central China mainly is anthropophagic, but in Liaoning preferred animal's blood [11]. The above should be the key factors of influencing population genetic structure of *An. lesteri *in China.

## Conclusion

Levels of genetic variation within *An. lesteri *populations were higher than among them. There was an apparent segregation from Liaoning with the other populations using mtDNA-COII gene. The results of neutrality test suggested a large number of low-frequency mutations in populations and the populations were in expansion proceeding. While these results may suggest considerable levels of gene flow, other explanations such as the effect of historical population perturbations can also be hypothesized.

## Competing interests

The authors declare that they have no competing interests.

## Authors' contributions

YM conceived and designed the experiments. MY performed the experiments. MY and YM analyzed the data. MY and YM wrote the paper. JW Provided part of the material. All authors read and approved the manuscript.
